# *N*-*tert*-Butyl-2-[4-(dimethyl­amino)­phen­yl]imidazo[1,2-*a*]pyrazin-3-amine

**DOI:** 10.1107/S1600536813007861

**Published:** 2013-03-28

**Authors:** Zeenat Fatima, Thothadri Srinivasan, Suman Koorathota, Sathiah Thennarasu, Devadasan Velmurugan

**Affiliations:** a Centre of Advanced Study in Crystallography and Biophysics, University of Madras, Guindy Campus, Chennai 600 025, India; b Organic Chemistry Division, Central Leather Research Institute, Adyar, Chennai 600 020, India

## Abstract

In the title compound, C_18_H_23_N_5_, the imidazole ring makes a dihedral angles of 3.96 (8) and 19.02 (8)°, respectively, with the pyrazine and benzene rings while the dihedral angle between the pyrazine and benzene rings is 16.96 (7)°. In the crystal, mol­ecules are linked *via* N—H⋯N hydrogen bonds, forming chains along [010]. These chains are linked by C—H⋯N hydrogen bonds, forming two-dimensional networks lying parallel to (001).

## Related literature

For applications of the pyrazine ring system in drug development, see: Du *et al.* (2009 ▶); Dubinina *et al.* (2006 ▶); Ellsworth *et al.* (2007 ▶); Mukaiyama *et al.* (2007 ▶). For ongoing structural studies of heterocyclic N-containing derivatives, see: Nasir *et al.* (2010 ▶). For background to the fluorescence properties of compounds related to the title compound, see: Kawai *et al.* (2001 ▶); Abdullah (2005 ▶). For general background to the use of imidazole derivatives as drugs, see: Dooley *et al.* (1992 ▶); Jackson *et al.* (2000 ▶); Banfi *et al.* (2006 ▶). For a related structure, see: Ouzidan *et al.* (2011 ▶).
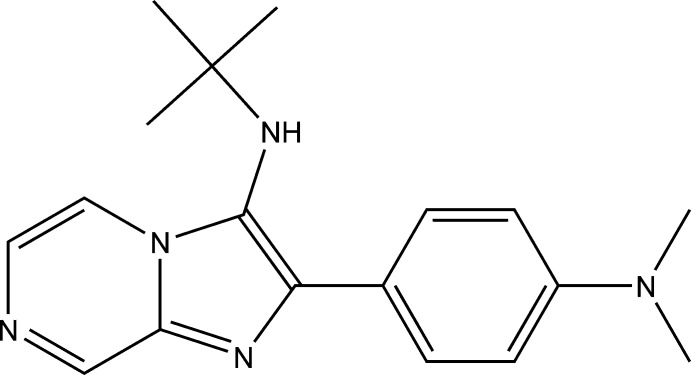


## Experimental

### 

#### Crystal data

C_18_H_23_N_5_
*M* *_r_* = 309.41
Orthorhombic, 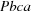

*a* = 12.1746 (11) Å
*b* = 13.9614 (13) Å
*c* = 20.2985 (19) Å
*V* = 3450.2 (6) Å^3^
*Z* = 8
Mo *K*α radiation
μ = 0.07 mm^−1^
*T* = 293 K
0.30 × 0.25 × 0.20 mm

#### Data collection

Bruker SMART APEXII area-detector diffractometer
Absorption correction: multi-scan (*SADABS*; Bruker, 2008 ▶) *T*_min_ = 0.978, *T*_max_ = 0.985
18314 measured reflections
4157 independent reflections
2964 reflections with *I* > 2σ(*I*)
*R*_int_ = 0.043

#### Refinement

*R*[*F* ^2^ > 2σ(*F* ^2^)] = 0.049
*wR*(*F* ^2^) = 0.148
*S* = 1.03
4157 reflections
218 parameters
H atoms treated by a mixture of independent and constrained refinement
Δρ_max_ = 0.32 e Å^−3^
Δρ_min_ = −0.25 e Å^−3^

### 

Data collection: *APEX2* (Bruker, 2008 ▶); cell refinement: *SAINT* (Bruker, 2008 ▶); data reduction: *SAINT*; program(s) used to solve structure: *SHELXS97* (Sheldrick, 2008 ▶); program(s) used to refine structure: *SHELXL97* (Sheldrick, 2008 ▶); molecular graphics: *ORTEP-3 for Windows* (Farrugia, 2012 ▶); software used to prepare material for publication: *SHELXL97* and *PLATON* (Spek, 2009 ▶).

## Supplementary Material

Crystal structure: contains datablock(s) global, I. DOI: 10.1107/S1600536813007861/su2577sup1.cif

Structure factors: contains datablock(s) I. DOI: 10.1107/S1600536813007861/su2577Isup2.hkl

Supplementary material file. DOI: 10.1107/S1600536813007861/su2577Isup3.cml

Additional supplementary materials:  crystallographic information; 3D view; checkCIF report

## Figures and Tables

**Table 1:** Hydrogen-bond geometry (Å, °)

*D*—H⋯*A*	*D*—H	H⋯*A*	*D*⋯*A*	*D*—H⋯*A*
N3—H3⋯N1^i^	0.873 (18)	2.201 (18)	3.0361 (18)	160.0 (14)
C11—H11⋯N4^ii^	0.93	2.53	3.428 (2)	162

Symmetry codes: (i) 
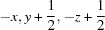
; (ii) 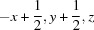.

**Fig. 1.**
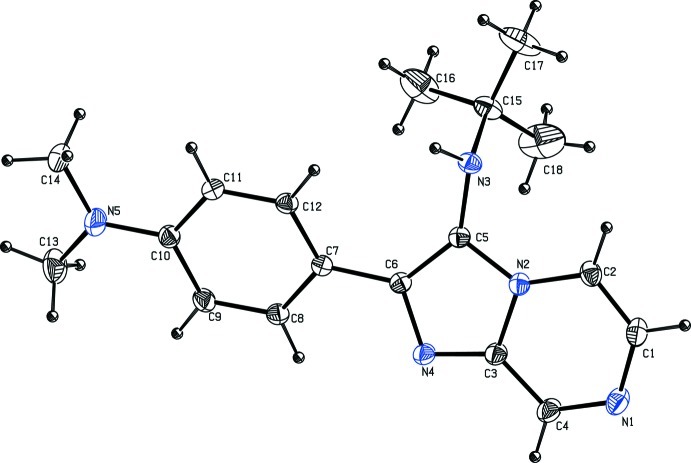
The molecular structure of the title molecule, with atom labelling. Displacement ellipsoids are drawn at the 30% probability level.

**Fig. 2.**
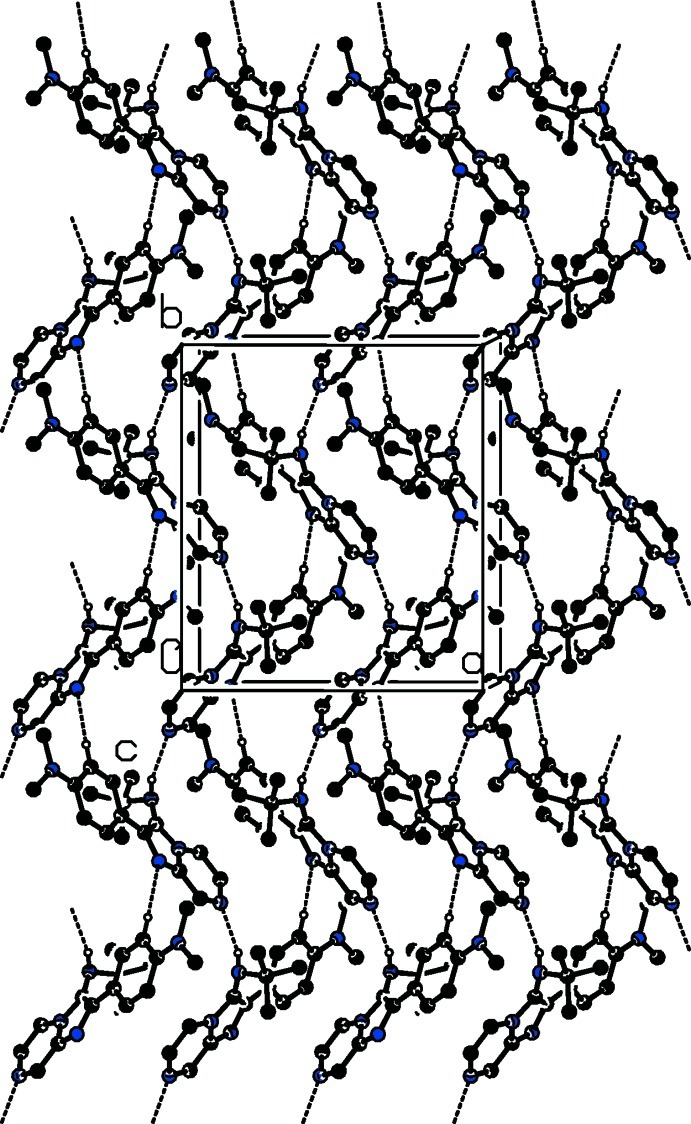
The crystal packing of the title compound viewed along the c axis. The hydrogen bonds are shown as dashed lines [see Table 1 for details; H-atoms not involved in hydrogen bonds have been omitted for clarity].

## supplementary crystallographic information

### Comment

The pyrazine ring system is a useful structural element in medicinal chemistry and has found broad applications in drug development which can be used as antiproliferative agents (Dubinina *et al.*, 2006), potent CXCR3 antagonists (Du *et al.*, 2009),CB1 antagonists (Ellsworth *et al.*, 2007) and c-Src inhibitory (Mukaiyama *et al.*, 2007). On-going structural studies of heterocyclic N-containing derivatives (Nasir *et al.*, 2010) are also motivated by an investigation of their fluorescence properties (Kawai *et al.*, 2001; Abdullah, 2005). For multidrug-resistant Tuberculosis (Dooley *et al.*,(1992)), antifungal and antimycobacterial activity (Banfi *et al.* 2006), and bactericidal effects (Jackson *et al.* 2000), the use of imidazole based compounds has been reported. In view of the different applications of this class of compounds, we have undertaken a single-crystal structure determination of the title compound.

In the titled compound, Fig. 1, the imidazole ring (N2/N4/C3/C5/C6) makes a dihedral angle of 3.96 (8)° with the pyrazine ring (N1/N2/C1-C4) and a dihedral angle of 19.02 (8)° with the benzene ring (C7-C12). The dihedral angle between the pyrazine ring and the benzene ring is 16.96 (7)°.The dimethylamine group (N5/C14/C15) attached with the benzene ring makes a dihedral angle of 8.84 (11)°.

In the crystal, molecules are linked via N-H···N hydrogen bonds forming chains along [010]. These chains are linked by C—H···N hydrogen bonds forming two-dimensional networks lying parallel to (001); see Table 1 and Fig. 2 for details.

### Experimental

2-aminoamidine (1.0 mmol) was placed in an oven-dried round bottom flask, dissolved in EtOH (5.0 mL) and stirred at room temperature. 4-N,N-dimethyl benzaldehyde (1.0 mmol), isocyanide (1.0 mmol) and Iodine (2.0 mol%) were added sequentially and the mixture stirred at room temperature for one hour. Progress of the reaction was monitored by TLC. When finished the reaction mixture was concentrated under reduced pressure and the crude product was partitioned between EtOAc and water. The organic phase was separated, and the residual product in the aqueous phase was extracted with EtOAc (2 × 10 mL). The combined organic extract was dried over anhydrous Na_2_SO_4_, filtered, concentrated and purified using column chromatography (silica gel 60-120 mesh, elutent: 2% EtOAc in hexane). Colourless block-like crystals, suitable for X-ray diffraction analysis, were obtained by slow evaporation of a solution of the title compound in ethanol at room temperature [M.p: 478 - 480 K; IR (KBr, cm^-1^): 3259 (NH)]

### Refinement

The NH H atom was located in a difference Fourier map and freely refined. The C-bound H atoms were placed in calculated positions and refined in the riding model: C—H = 0.93 - 1.08 Å with *U*_iso_(H) = 1.5*U*_eq_(C-methyl) and = 1.2*U*_eq_(C) for other H atoms.

### Crystal data

**Table:**

C_18_H_23_N_5_	*F*(000) = 1328
*M**_r_* = 309.41	*D*_x_ = 1.191 Mg m^−^^3^
Orthorhombic, *P**b**c**a*	Mo *K*α radiation, λ = 0.71073 Å
Hall symbol: -P 2ac 2ab	Cell parameters from 4157 reflections
*a* = 12.1746 (11) Å	θ = 2.0–28.3°
*b* = 13.9614 (13) Å	µ = 0.07 mm^−^^1^
*c* = 20.2985 (19) Å	*T* = 293 K
*V* = 3450.2 (6) Å^3^	Block, colourless
*Z* = 8	0.30 × 0.25 × 0.20 mm

### Data collection

**Table:**

Bruker SMART APEXII area-detector diffractometer	4157 independent reflections
Radiation source: fine-focus sealed tube	2964 reflections with *I* > 2σ(*I*)
Graphite monochromator	*R*_int_ = 0.043
ω and φ scans	θ_max_ = 28.3°, θ_min_ = 2.0°
Absorption correction: multi-scan (*SADABS*; Bruker, 2008)	*h* = −15→16
*T*_min_ = 0.978, *T*_max_ = 0.985	*k* = −18→17
18314 measured reflections	*l* = −16→27

### Refinement

**Table:**

Refinement on *F*^2^	Secondary atom site location: difference Fourier map
Least-squares matrix: full	Hydrogen site location: inferred from neighbouring sites
*R*[*F*^2^ > 2σ(*F*^2^)] = 0.049	H atoms treated by a mixture of independent and constrained refinement
*wR*(*F*^2^) = 0.148	*w* = 1/[σ^2^(*F*_o_^2^) + (0.0723*P*)^2^ + 0.8061*P*] where *P* = (*F*_o_^2^ + 2*F*_c_^2^)/3
*S* = 1.03	(Δ/σ)_max_ < 0.001
4157 reflections	Δρ_max_ = 0.32 e Å^−^^3^
218 parameters	Δρ_min_ = −0.25 e Å^−^^3^
0 restraints	Extinction correction: *SHELXL97* (Sheldrick, 2008), Fc^*^=kFc[1+0.001xFc^2^λ^3^/sin(2θ)]^-1/4^
Primary atom site location: structure-invariant direct methods	Extinction coefficient: 0.0055 (8)

### Special details

**Table:**

Geometry. All esds (except the esd in the dihedral angle between two l.s. planes) are estimated using the full covariance matrix. The cell esds are taken into account individually in the estimation of esds in distances, angles and torsion angles; correlations between esds in cell parameters are only used when they are defined by crystal symmetry. An approximate (isotropic) treatment of cell esds is used for estimating esds involving l.s. planes.
Refinement. Refinement of F^2^ against ALL reflections. The weighted R-factor wR and goodness of fit S are based on F^2^, conventional R-factors R are based on F, with F set to zero for negative F^2^. The threshold expression of F^2^ > 2sigma(F^2^) is used only for calculating R-factors(gt) etc. and is not relevant to the choice of reflections for refinement. R-factors based on F^2^ are statistically about twice as large as those based on F, and R- factors based on ALL data will be even larger.

### Fractional atomic coordinates and isotropic or equivalent isotropic displacement parameters (Å^2^)

**Table:**

	*x*	*y*	*z*	*U*_iso_*/*U*_eq_	
C1	−0.08078 (14)	−0.06634 (12)	0.30835 (9)	0.0504 (4)	
H1	−0.1275	−0.0743	0.3442	0.060*	
C2	−0.01406 (13)	0.01078 (11)	0.30790 (8)	0.0435 (4)	
H2	−0.0140	0.0542	0.3427	0.052*	
C3	0.04911 (13)	−0.03960 (10)	0.20176 (7)	0.0402 (3)	
C4	−0.01999 (15)	−0.11940 (11)	0.20827 (9)	0.0506 (4)	
H4	−0.0206	−0.1646	0.1746	0.061*	
C5	0.12824 (11)	0.09448 (10)	0.23825 (7)	0.0343 (3)	
C6	0.16191 (11)	0.07218 (10)	0.17397 (7)	0.0340 (3)	
C7	0.23754 (11)	0.12262 (10)	0.12939 (7)	0.0337 (3)	
C8	0.28253 (13)	0.07449 (10)	0.07535 (7)	0.0397 (3)	
H8	0.2628	0.0110	0.0680	0.048*	
C9	0.35502 (14)	0.11775 (12)	0.03267 (7)	0.0455 (4)	
H9	0.3843	0.0825	−0.0020	0.055*	
C10	0.38571 (12)	0.21406 (11)	0.04048 (7)	0.0405 (3)	
C11	0.33674 (12)	0.26364 (11)	0.09300 (7)	0.0396 (3)	
H11	0.3524	0.3283	0.0988	0.047*	
C12	0.26608 (12)	0.21882 (10)	0.13617 (7)	0.0375 (3)	
H12	0.2365	0.2538	0.1709	0.045*	
C13	0.51820 (19)	0.20205 (17)	−0.05008 (11)	0.0752 (6)	
H13A	0.5585	0.1523	−0.0281	0.113*	
H13B	0.5684	0.2428	−0.0734	0.113*	
H13C	0.4674	0.1740	−0.0806	0.113*	
C14	0.48781 (18)	0.35712 (15)	0.00500 (10)	0.0670 (5)	
H14A	0.4222	0.3952	0.0070	0.101*	
H14B	0.5314	0.3768	−0.0320	0.101*	
H14C	0.5292	0.3657	0.0448	0.101*	
C15	0.24118 (15)	0.15682 (14)	0.33195 (8)	0.0528 (4)	
C16	0.34950 (17)	0.1809 (3)	0.29967 (13)	0.1029 (10)	
H16A	0.3616	0.1387	0.2631	0.154*	
H16B	0.4078	0.1734	0.3311	0.154*	
H16C	0.3479	0.2460	0.2843	0.154*	
C17	0.2179 (2)	0.2255 (2)	0.38729 (11)	0.0970 (9)	
H17A	0.2182	0.2898	0.3707	0.146*	
H17B	0.2733	0.2190	0.4206	0.146*	
H17C	0.1472	0.2114	0.4059	0.146*	
C18	0.2491 (3)	0.0546 (2)	0.35636 (17)	0.1255 (13)	
H18A	0.1877	0.0409	0.3845	0.188*	
H18B	0.3162	0.0464	0.3806	0.188*	
H18C	0.2484	0.0116	0.3195	0.188*	
N1	−0.08393 (13)	−0.13359 (9)	0.25942 (8)	0.0540 (4)	
N2	0.05405 (10)	0.02334 (8)	0.25439 (6)	0.0364 (3)	
N3	0.14805 (10)	0.16613 (9)	0.28437 (6)	0.0393 (3)	
N4	0.11341 (11)	−0.01085 (9)	0.15268 (6)	0.0417 (3)	
N5	0.45877 (13)	0.25768 (12)	−0.00212 (7)	0.0601 (4)	
H3	0.1442 (13)	0.2223 (13)	0.2656 (8)	0.042 (4)*	

### Atomic displacement parameters (Å^2^)

**Table:**

	*U*^11^	*U*^22^	*U*^33^	*U*^12^	*U*^13^	*U*^23^
C1	0.0562 (10)	0.0418 (9)	0.0532 (9)	0.0031 (7)	0.0173 (8)	0.0078 (7)
C2	0.0505 (9)	0.0407 (8)	0.0394 (7)	0.0067 (7)	0.0083 (7)	0.0017 (6)
C3	0.0444 (8)	0.0323 (7)	0.0440 (8)	0.0002 (6)	0.0063 (7)	−0.0035 (6)
C4	0.0567 (10)	0.0364 (8)	0.0586 (10)	−0.0068 (7)	0.0142 (8)	−0.0076 (7)
C5	0.0361 (7)	0.0293 (7)	0.0375 (7)	0.0033 (6)	−0.0007 (6)	0.0002 (5)
C6	0.0340 (7)	0.0296 (7)	0.0385 (7)	0.0035 (5)	0.0007 (6)	−0.0020 (5)
C7	0.0322 (7)	0.0343 (7)	0.0345 (7)	0.0021 (5)	−0.0015 (5)	−0.0002 (6)
C8	0.0467 (8)	0.0332 (7)	0.0392 (7)	−0.0001 (6)	0.0016 (6)	−0.0043 (6)
C9	0.0532 (9)	0.0448 (9)	0.0384 (7)	0.0022 (7)	0.0086 (7)	−0.0059 (7)
C10	0.0369 (7)	0.0467 (9)	0.0379 (7)	−0.0026 (6)	−0.0001 (6)	0.0016 (6)
C11	0.0402 (7)	0.0366 (8)	0.0419 (7)	−0.0045 (6)	−0.0010 (6)	−0.0017 (6)
C12	0.0392 (7)	0.0352 (7)	0.0379 (7)	0.0011 (6)	0.0010 (6)	−0.0056 (6)
C13	0.0726 (14)	0.0869 (16)	0.0662 (12)	0.0015 (11)	0.0331 (11)	0.0044 (11)
C14	0.0725 (13)	0.0677 (12)	0.0610 (11)	−0.0256 (10)	0.0065 (10)	0.0113 (9)
C15	0.0494 (9)	0.0655 (11)	0.0434 (8)	0.0067 (8)	−0.0102 (7)	−0.0113 (8)
C16	0.0460 (12)	0.188 (3)	0.0746 (14)	0.0117 (15)	−0.0093 (11)	−0.0300 (17)
C17	0.0787 (15)	0.146 (3)	0.0659 (13)	0.0234 (16)	−0.0176 (12)	−0.0533 (15)
C18	0.147 (3)	0.091 (2)	0.138 (3)	0.0072 (19)	−0.092 (2)	0.0259 (18)
N1	0.0589 (9)	0.0375 (7)	0.0657 (9)	−0.0044 (6)	0.0185 (7)	0.0017 (7)
N2	0.0390 (6)	0.0311 (6)	0.0392 (6)	0.0031 (5)	0.0039 (5)	0.0009 (5)
N3	0.0445 (7)	0.0354 (7)	0.0378 (6)	0.0047 (5)	−0.0024 (5)	−0.0055 (5)
N4	0.0472 (7)	0.0340 (6)	0.0439 (7)	−0.0049 (5)	0.0072 (6)	−0.0060 (5)
N5	0.0613 (9)	0.0632 (9)	0.0557 (8)	−0.0138 (8)	0.0211 (7)	−0.0013 (7)

### Geometric parameters (Å, º)

**Table:**

C1—C2	1.349 (2)	C11—H11	0.9300
C1—N1	1.367 (2)	C12—H12	0.9300
C1—H1	0.9300	C13—N5	1.440 (2)
C2—N2	1.3778 (19)	C13—H13A	0.9600
C2—H2	0.9300	C13—H13B	0.9600
C3—N4	1.3291 (19)	C13—H13C	0.9600
C3—N2	1.3847 (18)	C14—N5	1.440 (2)
C3—C4	1.402 (2)	C14—H14A	0.9600
C4—N1	1.313 (2)	C14—H14B	0.9600
C4—H4	0.9300	C14—H14C	0.9600
C5—N2	1.3819 (18)	C15—N3	1.495 (2)
C5—N3	1.3912 (18)	C15—C17	1.504 (3)
C5—C6	1.4027 (19)	C15—C16	1.510 (3)
C6—N4	1.3709 (18)	C15—C18	1.514 (3)
C6—C7	1.4706 (19)	C16—H16A	0.9600
C7—C12	1.394 (2)	C16—H16B	0.9600
C7—C8	1.398 (2)	C16—H16C	0.9600
C8—C9	1.376 (2)	C17—H17A	0.9600
C8—H8	0.9300	C17—H17B	0.9600
C9—C10	1.405 (2)	C17—H17C	0.9600
C9—H9	0.9300	C18—H18A	0.9600
C10—N5	1.382 (2)	C18—H18B	0.9600
C10—C11	1.404 (2)	C18—H18C	0.9600
C11—C12	1.378 (2)	N3—H3	0.873 (17)
			
C2—C1—N1	124.07 (15)	N5—C14—H14A	109.5
C2—C1—H1	118.0	N5—C14—H14B	109.5
N1—C1—H1	118.0	H14A—C14—H14B	109.5
C1—C2—N2	118.00 (14)	N5—C14—H14C	109.5
C1—C2—H2	121.0	H14A—C14—H14C	109.5
N2—C2—H2	121.0	H14B—C14—H14C	109.5
N4—C3—N2	111.17 (13)	N3—C15—C17	106.49 (15)
N4—C3—C4	131.60 (14)	N3—C15—C16	111.26 (15)
N2—C3—C4	117.23 (13)	C17—C15—C16	110.3 (2)
N1—C4—C3	123.38 (15)	N3—C15—C18	109.98 (17)
N1—C4—H4	118.3	C17—C15—C18	111.6 (2)
C3—C4—H4	118.3	C16—C15—C18	107.2 (2)
N2—C5—N3	118.08 (12)	C15—C16—H16A	109.5
N2—C5—C6	104.60 (12)	C15—C16—H16B	109.5
N3—C5—C6	137.30 (13)	H16A—C16—H16B	109.5
N4—C6—C5	110.80 (12)	C15—C16—H16C	109.5
N4—C6—C7	118.73 (12)	H16A—C16—H16C	109.5
C5—C6—C7	130.48 (13)	H16B—C16—H16C	109.5
C12—C7—C8	116.29 (13)	C15—C17—H17A	109.5
C12—C7—C6	123.82 (12)	C15—C17—H17B	109.5
C8—C7—C6	119.86 (13)	H17A—C17—H17B	109.5
C9—C8—C7	122.29 (14)	C15—C17—H17C	109.5
C9—C8—H8	118.9	H17A—C17—H17C	109.5
C7—C8—H8	118.9	H17B—C17—H17C	109.5
C8—C9—C10	121.30 (14)	C15—C18—H18A	109.5
C8—C9—H9	119.3	C15—C18—H18B	109.5
C10—C9—H9	119.3	H18A—C18—H18B	109.5
N5—C10—C11	122.09 (14)	C15—C18—H18C	109.5
N5—C10—C9	121.49 (14)	H18A—C18—H18C	109.5
C11—C10—C9	116.41 (13)	H18B—C18—H18C	109.5
C12—C11—C10	121.61 (14)	C4—N1—C1	117.02 (14)
C12—C11—H11	119.2	C2—N2—C5	132.20 (13)
C10—C11—H11	119.2	C2—N2—C3	120.09 (13)
C11—C12—C7	122.01 (13)	C5—N2—C3	107.56 (12)
C11—C12—H12	119.0	C5—N3—C15	120.24 (12)
C7—C12—H12	119.0	C5—N3—H3	110.0 (11)
N5—C13—H13A	109.5	C15—N3—H3	113.6 (11)
N5—C13—H13B	109.5	C3—N4—C6	105.83 (12)
H13A—C13—H13B	109.5	C10—N5—C14	121.33 (15)
N5—C13—H13C	109.5	C10—N5—C13	120.58 (16)
H13A—C13—H13C	109.5	C14—N5—C13	117.68 (16)
H13B—C13—H13C	109.5		
			
N1—C1—C2—N2	1.0 (3)	C1—C2—N2—C5	177.97 (15)
N4—C3—C4—N1	−175.41 (17)	C1—C2—N2—C3	3.0 (2)
N2—C3—C4—N1	4.2 (3)	N3—C5—N2—C2	5.3 (2)
N2—C5—C6—N4	−2.09 (16)	C6—C5—N2—C2	−173.15 (14)
N3—C5—C6—N4	179.96 (16)	N3—C5—N2—C3	−179.30 (12)
N2—C5—C6—C7	178.06 (14)	C6—C5—N2—C3	2.27 (15)
N3—C5—C6—C7	0.1 (3)	N4—C3—N2—C2	174.29 (13)
N4—C6—C7—C12	160.24 (13)	C4—C3—N2—C2	−5.4 (2)
C5—C6—C7—C12	−19.9 (2)	N4—C3—N2—C5	−1.79 (17)
N4—C6—C7—C8	−17.7 (2)	C4—C3—N2—C5	178.51 (14)
C5—C6—C7—C8	162.11 (14)	N2—C5—N3—C15	93.24 (17)
C12—C7—C8—C9	2.9 (2)	C6—C5—N3—C15	−89.0 (2)
C6—C7—C8—C9	−178.96 (14)	C17—C15—N3—C5	−161.45 (18)
C7—C8—C9—C10	−1.5 (2)	C16—C15—N3—C5	78.3 (2)
C8—C9—C10—N5	179.77 (15)	C18—C15—N3—C5	−40.4 (2)
C8—C9—C10—C11	−1.3 (2)	N2—C3—N4—C6	0.46 (17)
N5—C10—C11—C12	−178.37 (15)	C4—C3—N4—C6	−179.89 (17)
C9—C10—C11—C12	2.7 (2)	C5—C6—N4—C3	1.04 (16)
C10—C11—C12—C7	−1.3 (2)	C7—C6—N4—C3	−179.08 (13)
C8—C7—C12—C11	−1.5 (2)	C11—C10—N5—C14	−0.5 (3)
C6—C7—C12—C11	−179.53 (14)	C9—C10—N5—C14	178.34 (17)
C3—C4—N1—C1	−0.5 (3)	C11—C10—N5—C13	171.93 (17)
C2—C1—N1—C4	−2.2 (3)	C9—C10—N5—C13	−9.2 (3)

### Hydrogen-bond geometry (Å, º)

**Table:**

*D*—H···*A*	*D*—H	H···*A*	*D*···*A*	*D*—H···*A*
N3—H3···N1^i^	0.873 (18)	2.201 (18)	3.0361 (18)	160.0 (14)
C11—H11···N4^ii^	0.93	2.53	3.428 (2)	162

Symmetry codes: (i) −*x*, *y*+1/2, −*z*+1/2; (ii) −*x*+1/2, *y*+1/2, *z*.
